# The impact of a social prescribing service on patients in primary care: a mixed methods evaluation

**DOI:** 10.1186/s12913-017-2778-y

**Published:** 2017-12-19

**Authors:** Dawn Carnes, Ratna Sohanpal, Caroline Frostick, Sally Hull, Rohini Mathur, Gopalakrishnan Netuveli, Jin Tong, Patrick Hutt, Marcello Bertotti

**Affiliations:** 1 Queen Mary University of London, Barts and The London School of Medicine and Dentistry, Centre for Primary Care and Public Health, 58 Turner St, London, E1 2AB UK; 2 University of Applied Sciences Western Switzerland, School of Health Sciences, Route des Cliniques 15, 1700 Fribourg, Switzerland; 3 University of East London, Institute for Health and Human Development, Water Lane, Stratford, London, E15 4LZ UK; 4 City and Hackney Clinical Commissioning Group, Queensbridge Group General Practice, 24 Holly Street, London, E8 3XP UK

**Keywords:** Mixed methods, Evaluation, Social prescribing, Primary care

## Abstract

**Background:**

Social prescribing is targeted at isolated and lonely patients. Practitioners and patients jointly develop bespoke well-being plans to promote social integration and or social reactivation. Our aim was to investigate: whether a social prescribing service could be implemented in a general practice (GP) setting and to evaluate its effect on well-being and primary care resource use.

**Methods:**

We used a mixed method evaluation approach using patient surveys with matched control groups and a qualitative interview study. The study was conducted in a mixed socio-economic, multi-ethnic, inner city London borough with socially isolated patients who frequently visited their GP. The intervention was implemented by ‘social prescribing coordinators’. Outcomes of interest were psychological and social well-being and health care resource use.

**Results:**

At 8 months follow-up there were no differences between patients referred to social prescribing and the controls for general health, depression, anxiety and ‘positive and active engagement in life’. Social prescribing patients had high GP consultation rates, which fell in the year following referral. The qualitative study indicated that most patients had a positive experience with social prescribing but the service was not utilised to its full extent.

**Conclusion:**

Changes in general health and well-being following referral were very limited and comprehensive implementation was difficult to optimise. Although GP consultation rates fell, these may have reflected regression to the mean rather than changes related to the intervention. Whether social prescribing can contribute to the health of a nation for social and psychological wellbeing is still to be determined.

## Background

Since the 1990s there has been a shift from the concept of the biomedical health care model to the biopsychosocial model of understanding health states and disease, particularly for non-communicable chronic illnesses such as back and neck pain [1]. In the last few years there has been an emergence of interventions focusing on the social component of care, such as social prescribing, art on prescription, exercise/physical activity on prescription, walking groups and the introduction of health trainers, with some evidence for behaviour change [2–4]. These aim to help people manage their chronic condition, prevent more serious health problems developing, and contribute to addressing health inequalities by building social support networks.

**Fig. 1:**
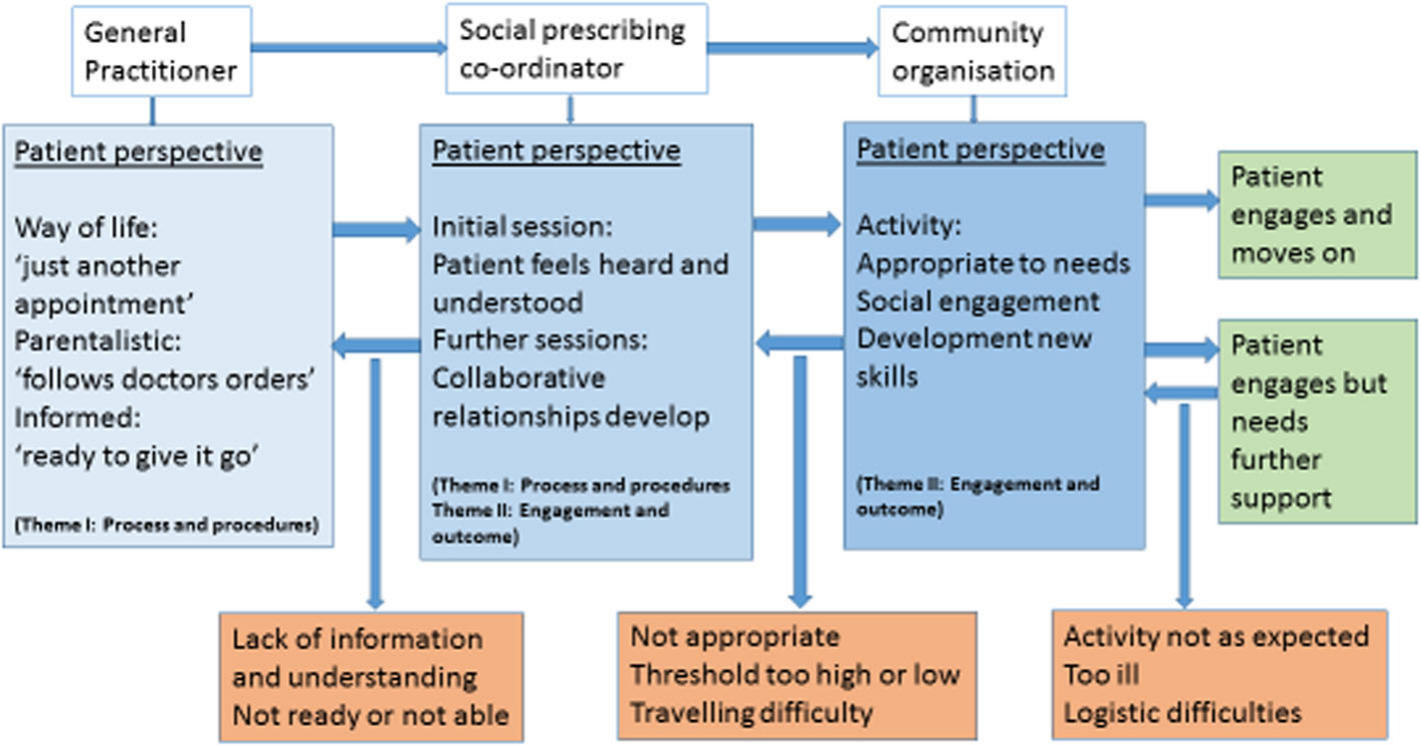
Description of barriers and facilitators to social engagement via the social prescribing service

The emergence of these interventions are in part due to the aging population, increases in chronic conditions, levels of social isolation and the growing burden of providing health care [5]. There is scope for providing new and innovative interventions to promote the self-management of chronic conditions potentially reducing the need for physician led care. Despite the increase in socially oriented health services, their effectiveness remains uncertain: a review of 12 evaluations of UK social prescribing services showed that the rigour of evaluations was limited and that none of the evaluations had an adequate control group [6]. However some of the service evaluation reports indicated some beneficial changes in anxiety, depression, wellbeing, social isolation and general practice attendance [7, 8].

There is no standard definition of social prescribing but we describe it as: a non-medical referral, or linking service, to help people identify their social needs and develop ‘well-being’ action plans to promote, establish or re-establish integration and support in their communities, with the aim of improving personal wellbeing.

In January 2014 the London Borough of City and Hackney Clinical Commissioning Group (CCG) commissioned a pilot project for a social prescribing service in three areas comprising 22 primary care general practices. The aim of the social prescribing service was to improve patient well-being and increase personal self-efficacy shown by a reduction in primary health care resource use.

The aim of the evaluation was twofold: i) to assess the effect of the service on mental wellbeing and primary health care resource use and ii) to assess the whether the service could be implemented as intended.

The aim of this paper is to present data about the effect of the service on the people referred and the implementation of the service from a patient perspective.

## Methods

We used a mixed methods approach to evaluate the service and test its effect on patients. For the service evaluation we monitored activity in the service and we interviewed patients to explore their views and experiences of the service. To compare the effect on patient reported health related outcomes we used a matched controlled group to assess ‘non-exposed’ patients using a postal questionnaire survey. For health care resource use we searched electronic patient records and compared those referred into social prescribing with a propensity matched control group. The evaluation period was from the service inception, Feb 1st 2014 to January 31st 2016.

### The setting

The social prescribing service was piloted in the London Borough of City and Hackney which is characterised by an extreme range of socio-economic deprivation and affluence and a considerable ethnic mix [9].

Three areas in the borough were included and were assigned a social prescribing coordinator. The coordinators were trained in social work and employed by a managing third sector (not-for-profit) organisation commissioned to implement the service. Three social prescribing coordinators were appointed and worked in the 22 GP surgeries enrolled.

### Population

The population of interest were patients in general practices who were frequent attenders and, or socially isolated. People were not referred if they were in acute crisis, at risk to self and/or others, had uncontrolled addictions or uncontrolled mental health problems.

### The social prescribing service

Patients were referred to a Social prescribing coordinator. At the first meeting with the coordinator, the patients discussed their personal circumstances and if possible a mutually determined well-being action plan was devised. The action plan contained goals for improving patient wellbeing, in some cases this involved referring patients to community organisations and services. If necessary a volunteer was assigned to help the patient achieve their goals. Volunteers were trained by the Social prescribing coordinators to assist in the delivery of the service and provide additional support to clients. Patients could receive up to six sessions with the social prescribing coordinator and as many contacts with the volunteer as required.

### Evaluation of effect of service on patients

- Patient reported mental wellbeing
  All patients referred to the social prescribing service were sent a questionnaire by the independent evaluation research team, prior to their first appointment (where possible) and after eight months. Patients were asked basic demographic information about: age, sex, employment status, education status, English language fluency, and living alone or not. We collected data about general health [10], wellbeing [11], anxiety and depression [12], number of regular activities, accident and emergency visits in last 3 months and positive and active engagement in life [13]. The eight month follow-up questionnaire also included questions about satisfaction.
  The control group consisted of a randomly selected group of patients from neighbouring areas not involved in the social prescribing scheme. People were selected and matched by: age, older than 23 and younger than 85 years, GP attendance (last 3 months) and at least one of the following: depression, anxiety, type 2 diabetes. Exclusion criteria were: palliative care and housebound.
  A sample of 3000 people were invited to take part in a questionnaire survey about their health and wellbeing with a view to getting around a 10% response rate (based on prior surveying experience in this population) to match the number of patients expected to be referred into the social prescribing service (300 over a 12 month period). These participants were given the same questionnaire as those referred into social prescribing and were followed up at 8 months.
  Analysis: We compared questionnaire mean scores from the social prescribing patients and the controls at baseline and at 8 months.
- Primary health care resource use
  Primary health care use data were collected electronically and anonymously from patient health care records. All GP referrals into the social prescribing scheme were flagged by a unique identification code. Matched controls were identified from the referring practice populations. For every patient referred, up to 20 matched controls with similar demographic characteristics were identified. Demographics used to match patients were: age, sex, ethnic group, general practice (by Index of Multiple Deprivation) and the presence of co-morbidities (cardiovascular disease, respiratory and mental health conditions). Using anonymised consultation and prescribing data we compared annual GP consultations and number of medications prescribed (antidepressants, antipsychotics, anxiolytics, nonsteroidal anti-inflammatory drugs and opioid analgesics) for the year prior and the year following the date of referral between social prescribing attenders and their matched controls.
  Analysis: We used non-parametric statistics and linear regression to compare the social prescribing group with the controls.

### Evaluation of the service from a patient perspective

In this report we present information about the patients and their perspectives of the service.

- Activity
  The commissioned service provider was requested to keep monthly records of the number of people referred into the service (by GP and practice), the sex, age, number and type of contacts with the social prescriber and volunteers and places that people were referred to in the community setting.
- Interviews with patients
  We used a phenomenological approach to capture patient experience, beliefs and opinions at one point in time. We tried to access patients who had fully engaged with the social prescribing service (2 or more contacts), partially engaged (1 contact) and those who did not engage at all (0 contacts). We randomly sampled 100 patients from each category to approach for interview. Subsequently we aimed to interview 20 patients by using purposive sampling to maximise the variety and range of patients interviewed in terms of sex, age and ethnicity.
  We conducted semi-structured interviews covering: lifestyle (to establish their levels of social isolation), the way they were referred into the service, what they knew about the service, their level of engagement in, and experience of the service and recommendations for the future. Four of the authors (DC, MB, CF, RS) conducted the interviews by telephone and face to face where possible. Copious notes were taken, interviews were not transcribed verbatim. Signed consent was obtained from each participant.
  Analysis: We familiarised ourselves with the content of the interviews and organised the data by responses to questions (the topic areas). By consensus we agreed on emerging themes and sub-themes. Data was aligned with each theme and sub-theme and any dissonant data or data left over was considered separately.

## Results

### Quantitative evaluation

Twenty-two general practices referred patients into the social prescribing service (range: patients per practice 1–108). The mean number of patients referred per month was 45 (range 25–59). A total of eighty-two community organisations were used in the delivery of the service, although most participants were sent to 10% of these. The community organisations were diverse and reflected the different interests of people, for example exercise classes, cookery lunch clubs, library visits, religious groups and ping pong. Nineteen volunteers were trained, 10 were used.

Of the patients referred (Table 1), 17% had more than one contact with the service, 14% had no contact at all and the remainder had one contact. Patient reasons for non-engagement following referral included: declined to participate, reason unknown, uncontactable, other commitments, ill health, moved away, unclear of reason for referral.

**Table 1:** Engagement in service (Feb 2014 – Mar 2015)

Consultations between patient and social prescribing coordinator/volunteer	Number (%) of people referred into social prescribing (*n* = 585)
No contact	81 (14)
Single consultation	405 (69)
Between 2 and 4 consultations	79 (14)
Between 5 and 6 consultations	20 (3)

### Patient reported outcomes

The questionnaire response rate for the social prescribing group at baseline was 39% (184/475) and at 8 months 38% (69/181). For the controls the response rate at baseline was 10% (302/3000) and at 8 months 42% (127/302) (Table 2).

**Table 2:** Demographic profile of participant and control patients responding to the survey at baseline

Characteristics	Control Group *n* = 302	Intervention Group *n* = 184	*P*-value
Age (Median (IQR))	58 (20)	56 (22)	0.376^a^
Gender (n (%))			
Female	164 (54)	103 (59)	
Male	137 (46)	72 (41)	0.354^b^
Ethnicity (n (%))			
White	170 (58)	88 (49)	
Non-White	123 (42)	90 (51)	0.070^b^
Living arrangement (n (%))			
Alone	106 (37)	101 (60)	
With others	180 (63)	66 (40)	< 0.001^b^
Work status (n (%))			
Not paid	153 (53)	162 (91)	
Paid	136 (47)	17 (9)	< 0.001^b^
Education (n (%))			
Up to 16 years	111 (39)	100 (58)	
17 years or above	175 (61)	72 (42)	< 0.001^b^

^a^Median test, ^b^Chi square test

The control and intervention groups differed in three ways, the control group were more likely to be living with others, in paid work and were in full time education for longer.

### Baseline data and change at 8 months 

There was no statistically significant difference in any outcome between baseline and 8 months (Tables 3 and 4).

**Table 3:** Comparison of outcome variables between baseline and 8 month follow-up

Outcomes	Control Group				Intervention Group			
	Baseline		Follow-up		Baseline		Follow-up	
	n	Mean (S.D.)	n	Mean (S.D.)	n	Mean (S.D.)	n	Mean (S.D.)
General health score^a^	296	3.3 (1.00)	127	3.3 (1.02)	184	2.8 (1.00)	65	2.7 (0.95)
HADS Anxiety score (range 0–21)^b^	287	8.1 (5.47)	124	7.6 (5.43)	175	11.3 (5.02)	63	11.2 (5.02)
HADS Depression score (scale 0–21)^b^	295	6.7 (5.22)	124	5.9 (5.22)	174	9.9 (5.08)	64	10.1 (5.06)
HADS score (scale 0–41)^b^	286	14.8 (9.88)	122	13.4 (9.99)	169	21.1 (9.57)	63	21.3 (9.36)
Wellbeing (past week) (range 0–6)	300	3.6 (1.52)	126	3.9 (1.44)	184	2.8 (1.47)	65	2.8 (1.44)
Active engagement in life score (scale 0–20)^c^	293	13.7 (3.92)	121	14.1 (3.89)	179	13.5 (3.88)	62	13.5 (3.83)
Number of regular activities (range 0–6)	302	2.8 (2.24)	126	2.9 (2.27)	184	1.9 (1.66)	43	1.3 (1.31)
A&E visits in past 3 months	289	0.3 (0.79)	121	0.5 (1.15)			47	0.3 (0.68)

^a^General health scores 1 = very bad; 5 = very good. ^b^Anxiety and depression Scores between 0 and 7 in both anxiety and depression scales are considered normal, with 8–10 borderline and 11 or over indicating clinical ‘caseness’. ^c^HeiQ Scale is between 5 and 20: 5 = poorly integrated; 20 = well integrated

**Table 4:** Effect of social prescribing on general and mental health, wellbeing and active living

Linear regression model on outcome differences (between baseline and follow-up) against treatment group		
Outcomes	Non-adjusted	Adjusted^a^
	Coef. (95% Conf. Interval)	Coef. (95% Conf. Interval)
General health score	−0.029 (−0.312, 0.253)	0.127 (−0.221, 0.475)
HADS Anxiety score (range 0–21)	−0.542 (−1.837, 0.752)	−0.119 (−0.847, 1.609)
HADS Depression score (range 0–21)	0.679 (−0.566, 1.924)	0.857 (−0.737, 2.451)
HADS score (range 0–41)	0.232 (−2.113, 2.577)	0.906 (−2.144, 3.957)
Wellbeing (past week) (range 0–6)	−0.089 (−0.569, 0.391)	−0.013 (−0.623, 0.596)
Active engagement in life score (range 0–20)	0.023 (−0.957, 1.004)	−0.073 (−1.278, 1.131)
Number of regular activities^b^	−0.856 (−1.518, −0.194)	−0.897 (−1.729, −0.065)

^a^Adjusted with control variables, including age, sex, ethnicity, work status and living arrangement

^b^ *p = 0.012* for non-adjusted model and *p = 0.035* for adjusted model

Both the social prescribing group and the control group showed positive changes in anxiety (though not depression) over the 8 months period. However, the control sample was in better mental health at baseline (Table 3).

The change in patient reported outcome scores for general health, depression, anxiety, wellbeing and active engagement in life were analysed using a linear regression model (Table 4). This type of analysis predicts what treatment effect our social prescribing service had on people in the study. Both the non-adjusted and adjusted models (taking into account the different demographic profiles) showed that the social prescribing service did not have any statistically significant effects on patients’ general and mental health, wellbeing and active living changes. However there was a reduction in the number of activities between baseline and follow-up indicating a negative effect.

### Health care resource use 

Across the participating general practices, the study identified 381 patients referral to social prescribing. For these 381 participants, 7540 controls, matched by age, sex, ethnicity and co-morbidities were identified (Table 5).

**Table 5:** Comparison of GP consultation and medication use before and after referral date between those referred to social prescribing and controls

Social prescribing	N control	Median (IQR)	N referred into social prescribing	Median (IQR)	Two-sample Wilcoxon rank-sum (Mann-Whitney) test for non-parametric data
Annual GP consultation rate before referral	7540	2.9 (0.6–5.8)	377	8.3 (5.8–12.1)	*p* < 0.001
Annual GP consultation rate after referral	7540	3.3 (0–6.4)	377	7.3 (4.7–10.7)	*p* < 0.001
Two-sample Wilcoxon rank-sum (Mann-Whitney) test for non-parametric data		*p* = 0.014		*p* = 0.001	
No. of medications 6 months before referral	7540	0 (0–1)	377	2 (1–3)	*p* < 0.001
No. of medications 6 months after referral	7540	0 (0–1)	377	2 (1–3)	*p* < 0.001
Two-sample Wilcoxon rank-sum (Mann-Whitney) test for non-parametric data		*p* = 0.022		*p* = 0.156	

The annual GP consultation rate in those referred to social prescribing was significantly higher than in controls both before and in the year following the date of referral to social prescribing. The GP consultation rate within controls was higher after their matched comparator referral date compared to before, whilst the GP consultation rate for those referred into social prescribing was lower after the referral date compared to before (Table 5).

The analysis showed that the number of medications prescribed to cases was significantly higher for those referred into social prescribing both before and after the intervention. The number of medications prescribed increased slightly in the controls after the referral date but the number of medications prescribed in those referred to social prescribing remained stable (Table 5).

### Qualitative evaluation

#### Satisfaction with the service

Most clients (55% 35/60)) were satisfied with the social prescribing service they received at 8 months, 70% (42/60) would have liked more information about the service and 62% (39/63) would recommend the service to others.

#### Patient interviews

Of the randomly selected 100 clients from the three different groups: full, partial and non-attenders, to be interviewed: fifteen people responded to the letters and consented to be interviewed. Of these, five people were available for interview. The remainder were too busy, non-contactable or did not want to participate. Examples for non-participation included: moved, unwell, in hospital.

As our sampling method only generated five interviews, we asked the managing organisation to contact an additional participants. This resulted in the completion of 15 additional interviews which were well balanced between sex, ethnicity and age (63% were aged 50 years or over) but all had engaged in the service. Of those interviewed two people had been invited to social prescribing but did not attend, six attended one or two sessions the remainder (7) three or more.

Two strong themes emerged from the data about: I) Processes and procedures and II) Engagement and outcomes. No dissonant data was found.

#### Theme I. Processes and procedures

Sub themes included i) patients being overwhelmed by their care provision and ii) appropriateness and timing of the referral. This included GP ‘parentalism’ and where patient’s thresholds of needs were too high (or low).

*“My GP knows me so well he probably just referred me because he thought it would be good for me”* (Pt partially engaged)

*“I had too many other things going on [family crises]”* (Pt not engaged)

Some interviewees were not sure what social prescribing was and who the service delivery organisation was, despite having been referred.

*“I have no idea who or what you are talking about, but sounds a good idea, I don’t know why I was referred……..”* (Pt not engaged)

Names used for social prescribing coordinators included: wellbeing coordinators, managing organisation support, social prescribers, counsellors, navigators, link workers, supporters, members of the general practice team.

The coordinators established themselves as part of the general practice services, in part because the one to one consultations that happened in the general practice surgeries. As a consequence users did not recognise the term social prescribing but only remembered their coordinators. This was mainly because the people we interviewed saw so many different health care professionals they had lost track of who they were seeing.

*“I don’t know who she was [in terms of health care professional]……I can’t remember her name…..errr but she was very nice”* (Pt engaged)

*“The problem is there are lots of services and lots of names, I get confused”* (Pt partially engaged)

#### Theme II. Engagement and outcome

The sub themes focused on the coordinator and patient relationship and understanding of the service. Where contact with wellbeing coordinators was established, this left a lasting impression either because expectations were surpassed or because expectations were unmet (often illustrating a lack of understanding about the service). The role of coordinators seemed to work best when they addressed some of the entrenched health and well-being issues patients had, illustrating that the role of a social prescribing coordinator was more than logistical coordination but important for facilitating the self-management of life skills and thus the health conditions. Wellbeing co-ordinators dealt with a range of needs from straightforward sign-posting to, what was in essence, a more intensive coaching-style intervention. Some of the most positive outcomes reported by patients resulted from experiencing sessions which allowed them the time to explore their situation more fully and work collaboratively to set realistic goals for the future.

*“It’s done me a world of good, taken me out of the house, given me a routine and given me a sense of purpose and …hope. It’s given me back my confidence”* (Pt engaged)

*“It [social prescribing] gave me the motivation to think I might be ready to go back to work”* (Pt engaged)

*“It [a voluntary organisation return to work scheme] allowed me to keep my hand in, so when I was ready to go back to work [this meant] I wouldn’t have not been working since 2012………….I’ve [now] got references and skills that are current”* (Pt engaged)

#### Overall interpretive analysis

Figure 1, illustrates the process of social prescribing in City and Hackney. It shows three phases in the process, first with the GP, then the social prescriber and finally the patient’s/client’s entry into the voluntary sector or the community. The ‘successful’ clients moved through each stage and emerged after exposure to all three stages as ready to move on or requiring further support. The reasons for not going through each stage are shown in the orange boxes and include: lack of understanding, lack of perceived need, overwhelmed by other health needs, logistical problems getting out and about.

## Discussion

All the participating general practices referred patients into the service. Of the patients referred 69% received at least one contact, either by telephone or face to face, only 17% received two or more contacts. This limited exposure to the service may partly explain the lack of impact on outcomes.

Those referred into social prescribing seemed to fit the referral criteria. They consulted more frequently and were prescribed more medication than the controls, and were significantly more likely to be living alone and unemployed. They were more anxious, depressed, they rated their general health and wellbeing worse than controls but interestingly their level of positive and active engagement in life were about the same.

There were no significant changes in general health, wellbeing, anxiety, depression, levels of positive and active engagement in life over time in either the social prescribing or the control groups. A finding which is difficult to explain is the reduction in number of activities in the intervention arm. In contrast the qualitative study showed there were strong and powerful narratives about the impact social prescribing had on some patients.

The consultation data needs interpreting with care because: i) there were a large number of controls, so a small rise in the median value for consultation rate over time for controls (2.9 to 3.3) was statistically significant and ii) the statistically significant drop in median GP consultation rate from 8.3 to 7.3 was in part, because GPs referred patients because they had higher than average rates of attendance (alongside perceived social isolation) so the identified changes may represent regression to the mean, rather than a change related to the efficacy of the intervention. Without evidence from a randomised controlled trial it would be premature to conclude that social prescribing reduced GP consultation rates.

### Strengths and limitations of this evaluation

The major strength of this evaluation is that it had two control groups: one for the comparison of patient reported outcomes by questionnaire, and the other for primary health care resource use using electronic patient records. This is the most comprehensive control group comparison to date.

A weakness however is the response rates, as with any evaluation of this nature in communities where English fluency and literacy is varied, it is difficult to collect data via postal questionnaires. The response rate at 8 months from those referred into social prescribing was only 14% (69/475). Furthermore, we do not have data about which clients actually went to which activities, organised by which voluntary organisations and how many times they went. The impact of the social prescribing service in the community organisations is not discernible but we do know that a large number of diverse organisations, activities and events were recommended.

### Findings in relation to other studies

The findings for social prescribing type of initiatives are comparable to other trials and evaluations of self-management programmes [14, 15] in that the evidence for effectiveness is inconsistent, small to moderate, at best, and only on some outcomes [6, 7, 16]. Health care resource use and subsequent evidence of reduction, and hence cost, remain powerful indicators for commissioners to fund these sorts of interventions. Two other studies reported promising data about health care resource use reduction as did we but whilst this data shows promise as with the other two studies the results have to be viewed with caution. Our qualitative study elicited strong positive narratives similar to case studies reported in other evaluations [17–19] but the quantitative data did not support or reflect the strength of these narratives throughout the whole referred group.

Due to the complex nature of these patients, long term multiple health states and social conditions, resolution perhaps is not the end goal but better quality of life and /or mental wellbeing. Given the discrepancy between the qualitative and quantitative literature on social prescribing, it could be that the standard health outcome measures do not capture the ‘non-health’ related outcomes that reflect patient priorities and their perspective of their own health and wellbeing [20]. Another point worth making, is that interventions of this nature that require the person’s active participation, engagement and commitment might be ‘exposure’ or ‘dose’ dependent, at present little is known about levels of patient exposure and intervention fidelity that might affect outcome [21–24].

### Implications for practice and research

Fidelity, making sure the service is delivered as it should be can be difficult, the problems encountered in the implementation of this social prescribing service have been experienced by others and recommendations to optimise service reported elsewhere [22]. From our evaluation experience we would emphasise the following:

1. The social prescribing has a patient recognisable identity
2. Coordinators are located in the GP surgery
3. Co-ordinators have non-clinical training, strong interpersonal and motivational skills.
4. Assessment of outcomes are those important to patients

Further research is needed from the GPs point of view. We propose that being able to share the needs of highly dependent patients with a social prescribing service is valuable in itself. Further work may need to be done to establish the right measurement tools and the appropriate timescale for data collection and a cluster randomised controlled trial with a full health economic analysis might provide more robust evidence for policy makers and commissioners thinking of this type of service provision. More work is also needed to ensure optimal delivery of social interventions to understand their potential effects. The concept of delivering social interventions on the theoretical assumption that building social self-efficacy can relieve congestion in the GP surgery may be misguided. Perhaps a better conception is to give value to self-efficacy and social capital and consider other mechanisms to reduce attendance at GP surgery.

## Conclusion

Changes in general health and well-being following referral were very limited. Comprehensive implementation was difficult to optimise and possibly explains the poor quantitative outcomes in comparison with the positive narratives reported by those fully engaging with the service. Although GP consultation rates fell, these may have reflected regression to the mean rather than changes related to the intervention. Social prescribing is still in relative infancy and the health benefit of social and psychological well-being as part of the overall health of a nation strategy is still to be determined.

## Abbreviations

A&E: Accident and emergency department
CCG: Clinical Commissioning group
GP: General practitioner
HADS: Hospital anxiety and depressions scale
NHS: National Health Service
UK: United Kingdom
